# Primary Care Clinic Approaches to Facilitating Patient Health Behavior Change in Alabama

**DOI:** 10.7759/cureus.55973

**Published:** 2024-03-11

**Authors:** Kyle R Distler, Marla Jo Lindsey, Mary Hinson Mims, Mary Ann Taylor, Joshua C Hollingsworth

**Affiliations:** 1 Preventive Medicine, Edward Via College of Osteopathic Medicine (VCOM-Auburn), Auburn, USA; 2 Psychiatry and Neuro-behavioral Sciences, Center for Institutional, Faculty, and Student Success, Edward Via College of Osteopathic Medicine (VCOM-Auburn), Auburn, USA; 3 Pharmacology, Edward Via College of Osteopathic Medicine (VCOM-Auburn), Auburn, USA

**Keywords:** health-related effects of physical activity, survey study, prevention in primary care, non-communicable chronic disease, preventative care, clinical practice survey, health behavior change

## Abstract

Background

Non-communicable chronic diseases (NCCDs), such as cardiovascular disease, diabetes, and cancer, are the leading cause of death and disability and the leading driver of healthcare costs in the U.S. It is estimated that 80% of chronic diseases and premature deaths are attributable to modifiable lifestyle factors related to smoking and alcohol intake, poor eating patterns, and physical inactivity. Inadequate sleep also plays a significant role. Among other directives, primary care providers (PCPs) have the opportunity to contribute to preventing and treating NCCD in their patients. Comprehensive, evidence-based behavioral counseling interventions are recommended to PCPs as a first-line approach to improving outcomes. However, presumably due to a lack of PCP time, training or resources, most patients report not receiving such services. Currently, the extent to which PCPs in Alabama offer or refer patients to health behavior change (HBC) services is unknown.

Objectives

This study aims to assess the following: (1) Alabama PCPs’ current approaches in facilitating patient HBC in the domains of eating patterns, physical activity, sleep, and stress and (2) the likelihood of the Alabama PCPs referring patients to virtual HBC programs, once developed by an osteopathic medical school in the state.

Methods

Data were collected from clinic personnel who were knowledgeable regarding the clinic’s approach to facilitating patient HBC via scripted telephone interviews and online surveys sent via email. The clinic list utilized for the study was derived from a list of VCOM-Auburn clinical preceptors. Primary care and specialty clinics were included. Data were analyzed descriptively to determine the number of clinics that (1) provide, recommend, or refer programs, services, or resources to patients to facilitate HBC related to eating patterns, physical activity, sleep, and stress management and (2) are likely to refer patients to free virtual HBC programs, once developed by an osteopathic medical school in the state.

Results

Of the 198 clinics that were contacted, 75 were excluded, 46 were “no response,” 53 agreed to participate, and 50 completed the survey. Of the 50 clinics that completed the survey, 33 indicated offering resources or referrals for diet, 29 stated they offered resources or referral services for physical activity, 33 indicated offering resources or referrals for sleep, and 28 indicated offering or recommending resources for stress management to patients. Most of the clinics (29/50) felt that their patients would benefit most from a program that facilitates improvement in eating patterns, and 41/50 clinics said that they are either “somewhat” or “extremely” likely to refer patients to a free VCOM-Auburn HBC program, once available.

Conclusions

Findings indicate that a significant percentage of PCP clinics are not offering HBC resources to patients and that most PCP clinics would consider referring patients to free VCOM-Auburn HBC programs, once available. Phone data were significantly different from email data. The primary limitations were a low response rate and potential response bias.

## Introduction

Non-communicable chronic diseases (NCCDs), such as cardiovascular disease, cancer, and diabetes, are the leading cause of death and disability and the leading driver of healthcare costs in the U.S. According to the Centers for Disease Control and Prevention (CDC), 60% of adults have NCCD, 40% have two or more, and 70% of deaths are attributable to NCCDs in the U.S. [1]. Cardiovascular diseases are the leading cause of death and account for the most significant health expenditure in the U.S., costing an estimated $363 billion annually in healthcare services, medications, and lost productivity [2]. Cancer, the second leading cause of death in the U.S., is estimated to cost more than $200 billion annually [3]. Diabetes, another leading NCCD, affects over 34 million Americans and incurs an estimated total economic cost of $327 billion per year [4]. It is estimated that 80% of NCCDs and premature deaths are attributable to modifiable lifestyle factors related to eating patterns, physical inactivity, sleep, and stress management [1].

A significant percentage of NCCDs and premature deaths are connected to modifiable lifestyle behaviors, primarily diet and physical activity. Poor eating patterns characterized by consuming high amounts of simple sugars, processed foods, and unhealthy fats increase the risk of many NCCDs, including obesity, diabetes, cancer, and heart disease [5]. Additionally, excessive alcohol consumption increases chronic disease and premature death risk. It is estimated that about 5% of deaths nationwide are due to chronic and acute effects of alcohol [6]. Limited physical activity is a leading risk factor for heart disease, diabetes, stroke, and certain types of cancer [7]. Furthermore, research shows strong associations between sleep disorders and hypertension, obesity, diabetes, pulmonary hypertension, accidents, heart disease, stroke, affective disorders, and neurodegenerative disorders [8]. Stress management is another critical lifestyle factor, as chronic stress increases blood pressure, heart disease and stroke risk, and other NCCDs [9,10]. These lifestyle factors together contribute significantly to NCCDs and premature deaths, indicating a drastic need for integrated lifestyle interventions in primary care. 

Eating pattern improvement can significantly reduce the risk of NCCDs and help treat them. Diets rich in fruits, vegetables, lean proteins, and whole grains with limited processed foods and added sugar are associated with a reduced risk of cardiovascular disease, diabetes, certain types of cancer, and other NCCDs [11]. The American Heart Association notes that adopting healthy eating patterns can lead to improvements in blood pressure, cholesterol levels, and body weight, all key risk factors for heart disease [12]. Similarly, the American Diabetes Association emphasizes the role of diet in both the prevention and management of diabetes, with a focus on controlling blood glucose levels through healthy eating [13]. Additionally, dietary improvements can have a broader impact on overall health by enhancing immune function and reducing inflammation, thereby offering protection against a wide range of NCCDs [14]. 

The shift from a sedentary lifestyle to engaging in low-to-moderate physical activity can have profound effects on health and significantly lower the risk of NCCDs. The World Health Organization (WHO) reports that inadequate physical activity is a leading risk factor for mortality, contributing to the rising incidence of heart disease, stroke, and certain types of cancer [7]. Regular physical activity, even at low-to-moderate intensities, has been shown to reduce the risk of NCCDs such as cardiovascular disease, type 2 diabetes, and certain cancers. The WHO highlights that as little as 150 minutes of moderate-intensity aerobic physical activity in a week can help reduce the risk of these diseases [7]. Similarly, engaging in regular physical activity can improve insulin sensitivity and glycemic control, playing a crucial role in the prevention and management of diabetes [15]. Increased physical activity can also help improve mental health, enhance immune function, and maintain a healthy body weight. 

Improving from poor or insufficient sleep to a pattern of more restful and adequate sleep can have a substantial impact on health and significantly decrease the risk of NCCDs. It is estimated that over a quarter of adults do not meet the American Academy of Sleep Medicine and the Sleep Research Society recommendation of at least seven hours of sleep per night [16]. Sleep helps regulate stress hormones and maintain a healthy balance of the body’s systems, and a sufficient amount of good quality sleep is vital for heart health [17]. Strong associations exist between sleep disorders, including obstructive sleep apnea, sleep onset or sleep maintenance insomnia, and NCCDs, including hypertension, obesity, diabetes, pulmonary hypertension, accidents, heart disease, stroke, affective disorders, and neurodegenerative disorders [8]. Further, the mental health and cognitive benefits provided by better sleep aid in the self-management of NCCDs. 

Transitioning from a state of high chronic stress to effective stress management can markedly improve health outcomes and reduce the risk of NCCDs. Poor stress management can negatively affect mental health, immune function, blood glucose, and blood pressure, and chronic stress is a known risk factor for numerous health conditions, including hypertension, heart disease, diabetes, and mental health disorders [9,18]. The American Psychological Association reports that effective stress management is crucial for reducing the risk of heart disease, as chronic stress contributes to elevated blood pressure and abnormal heart rhythms [18]. Studies show that poor eating patterns, physical inactivity, and insufficient sleep can increase stress, and the reverse is also true; high stress can negatively impact eating patterns, physical activity, and sleep [9,18]. Healthy lifestyle approaches to stress management, such as physical activity, cognitive behavioral therapy, mindfulness, and social support, should be promoted and utilized, while unhealthy approaches, such as substance use, should be avoided. For instance, it is estimated that 40% of smokers do so to manage stress, and the CDC estimates that one in five deaths in America is due to cigarette smoking [10]. While stress management counseling is not common with primary care providers (PCPs), it can be effective, along with psychological referrals and educational resources [19-22]. 

PCPs are responsible for preventing and treating NCCDs in their patients. Comprehensive, evidence-based behavioral counseling interventions are recommended to PCPs as a first-line approach to improving outcomes. PCPs can help educate and advise patients about healthy eating habits to prevent conditions such as heart disease and diabetes [11]. Primary care clinics are ideal to help motivate and educate individuals to adopt a more active lifestyle, which can reduce the risk of many NCCDs. Additionally, primary care physicians can screen for sleep disorders and provide guidance on sleep hygiene practices, an often overlooked but crucial aspect of overall health [8]. Effective stress management is another area where primary care interventions can have a significant impact, with physicians offering strategies and resources to help patients manage stress, thereby improving both mental and physical health outcomes [20,21]. For these behavior changes to be successful, primary care clinics must integrate multimodal approaches, including patient education, behavioral counseling and planning, and, when necessary, referrals to specialists. This comprehensive approach can lead to significant improvements in patient health outcomes, reducing the burden of NCCDs for the individual and society.

Despite guideline recommendations and known benefits, most patients report not receiving health behavior change (HBC) counseling and support [23]. A study published in the Journal of General Internal Medicine revealed that discussions about diet and exercise are infrequent during primary care visits, with less than a third of appointments including such conversations [24]. Similarly, sleep health and stress management are often overlooked, with PCPs either lacking sufficient time or resources to address these issues effectively [21,25]. Current payment models also play a role [26]. Furthermore, studies have shown that providers’ personal lifestyle affects their ability and willingness to counsel patients on HBC [21,25,27,28]. Regardless of the reason, PCP counseling and support for patient HBC appear to be underutilized. 

Currently, the extent to which PCPs in Alabama offer or refer patients to HBC services is unknown. The primary aims of this project were to assess the following: (1) how often and what resources or referral services, if any, primary care and specialty clinics in Alabama offered their patients and (2) how likely these clinics are to refer their patients to free virtual HBC programs, once available, that are currently under development by an osteopathic medical school in the state.

## Materials and methods

Built in Qualtrics, a survey was designed to assess healthcare clinics’ approaches to facilitating HBC in the domains of diet/eating patterns, exercise/physical activity, sleep, and stress management. For each of the four domains, the survey asked, “Currently, when you have a patient who is motivated to improve their [domain of interest], does your clinic have a program, service, or resource that you provide, recommend, or refer them to facilitate behavior change? This could be an in-person program, virtual web-based program, handout or pamphlet, a smartphone app, etc.” If the clinic responded, “yes,” then attempts were made to capture details regarding their approach. After responses for all four HBC domains were captured, the survey assessed the clinic’s likelihood of referring patients to free virtual HBC programs, once developed by an osteopathic medical school within the state. The survey also captured the HBC domain in which the clinic felt their patients would benefit most if given a free HBC program. Two versions of the survey were developed such that it could be administered over the phone to clinic personnel or completed digitally by clinic personnel at their convenience. 

Data were collected from clinic personnel (e.g., manager, nurse, and provider) who were knowledgeable about the clinic’s approach to facilitating HBC. Clinics were initially contacted via phone. When possible, the survey was administered at the time of first contact. When this was not possible, arrangements were made to administer the survey via phone sometime in the future, or the survey link was emailed to clinic personnel to be completed at their convenience. Clinics were contacted up to three times to solicit participation. 

Data were analyzed descriptively. Outcomes of interest included the following: (1) the percentage of clinics who provide, recommend, or refer programs, services, or resources to patients to facilitate HBC; (2) the details regarding the approaches used to facilitate HBC by participating clinics; (3) the likelihood of referring patients to free virtual HBC programs, once developed; and (4) the domain in which patients would derive the most benefit from a HBC program. As a secondary analysis, Fisher’s exact tests were used to assess for discrepancies in surveys completed over the phone versus those completed via email link. This study was approved by the VCOM-Auburn Institutional Review Board (IRB) on September 1, 2022 (protocol #2022-003).

## Results

**Figure 1:**
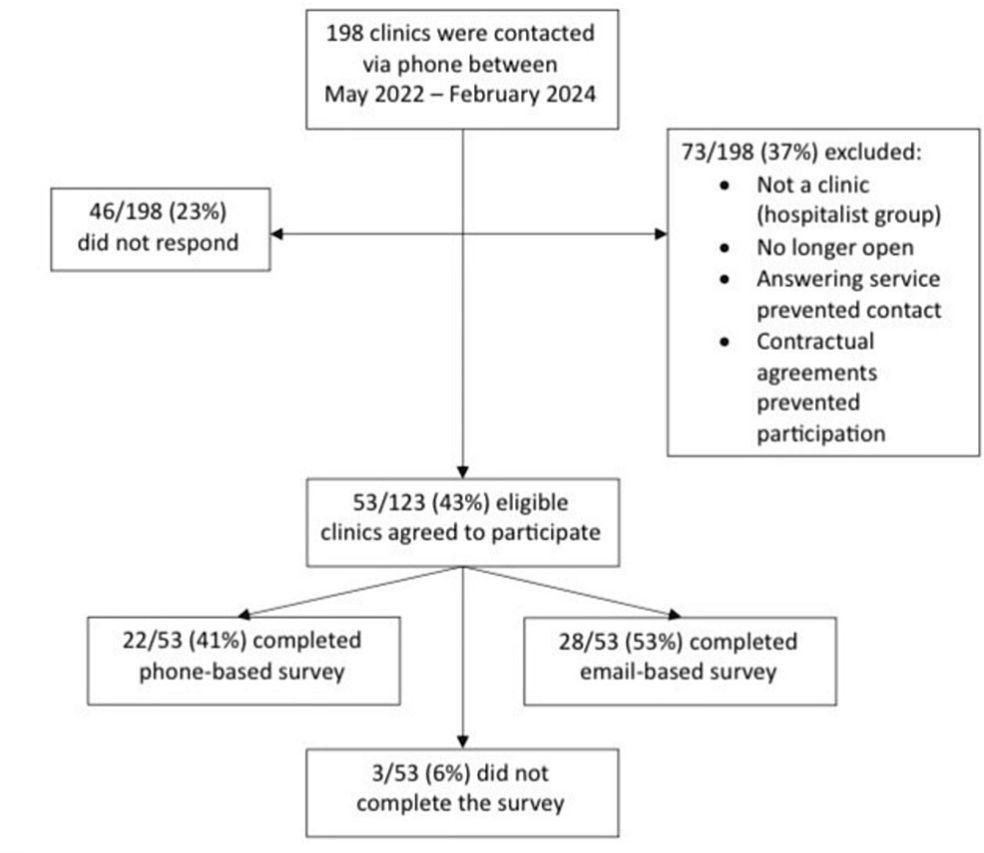
Study flow diagram

From May 2022 to February 2024, 198 clinics were contacted by phone. Seventy-five clinics were excluded due to one or more of the following reasons: the physician was no longer practicing, the clinic was a hospitalist group without an outpatient clinic, the clinic was unable to complete the survey due to contractual agreements, or, lastly, they were unable to be contacted due to answering service routing. As a result, 123 clinics met the inclusion criteria. No response was received from 46 clinics. Fifty-three clinics agreed to participate, and 50 (41%) completed the survey. Of those 50 clinics, 33 (66%) were family medicine, 10 (20%) were OBGYN clinics, two (4%) were cardiology clinics, and one clinic (2%) each for hematology, pain/wellness, pulmonology, psychological services, and rheumatology. In terms of clinic personnel completing the survey, 70% (35/50) were office/clinic managers, 12% (6/50) were head nurses (RN), 12% (6/50) were medical assistants, 4% were physicians (2/50), and 2% (1/50) was a clinic chief operating officer. Twenty-two surveys (44%) were completed via over-the-phone interviews, and the remaining 28 (56%) were completed digitally via email link (see Figure 1).

**Figure 2:**
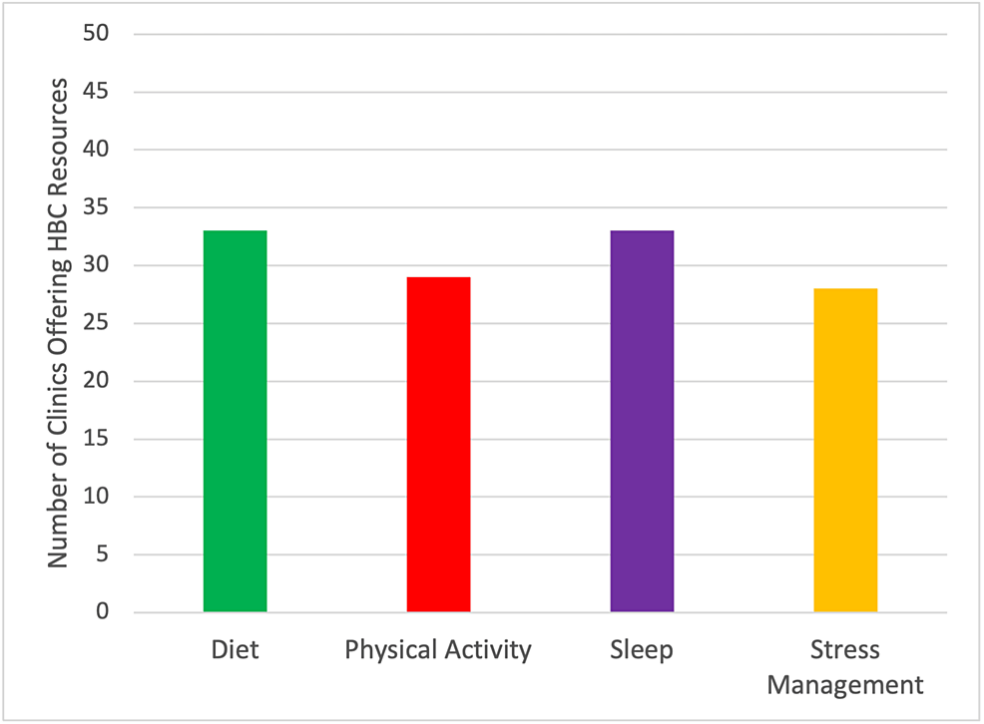
Number of clinics that indicated offering resources to facilitate health behavior change

**Figure 3:**
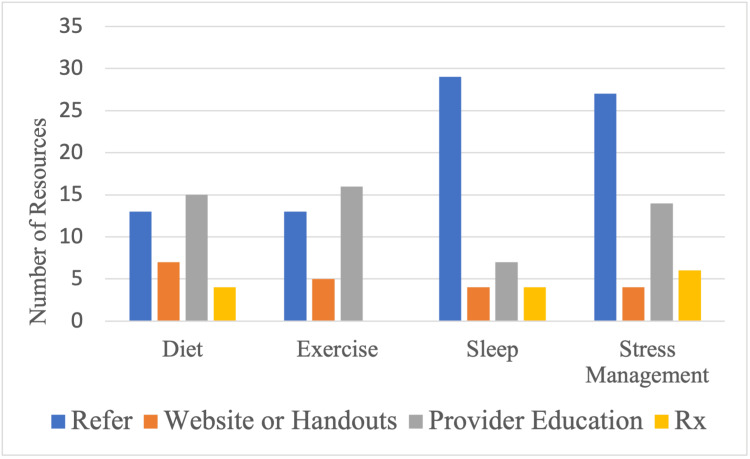
Types of resources indicated by clinics to facilitate health behavior change

Of the 50 clinics that completed the survey, 33 (66%) indicated offering resources or referrals for diet/eating pattern, 33 (66%) indicated offering resources or referrals for sleep, 28 (56%) indicated offering resources for stress management, and 29 (58%) indicated offering or recommending resources for exercise/physical activity to patients (Figure 2). Looking at clinic approaches to facilitating HBC change regarding eating patterns, further breakdown showed that 15/33 (45%) indicated using provider education, 13/33 (39%) indicated referring patients, 6/33 (18%) indicated providing websites of handouts, and 4/33 (12%) indicated prescribing medication. As for physical activity, 16/29 (55%) indicated using provider education, 13/29 (45%) indicated referring patients, and 5/29 (17%) indicated providing websites or handouts. For sleep, 29/33 (88%) indicated referring patients, 7/33 (21%) indicated using provider education, 4/33 (12%) indicated providing websites or handouts, and 4/33 (12%) indicated prescribing medication. Lastly, to facilitate HBC regarding stress management, 27/28 (96%) indicated referring patients, 14/28 (50%) indicated using provider education, 4/28 (14%) indicated providing websites or handouts, and 6/28 (21%) indicated prescribing medication (see Figure 3). The majority of clinics (29/50, 58%) felt that their patients would benefit most from a program focused on facilitating HBC in the domain of diet/eating patterns. Over 80% (41/50) of clinics said that they are either “somewhat” or “extremely” likely to refer patients to a free virtual HBC program, once available.

**Figure 4:**
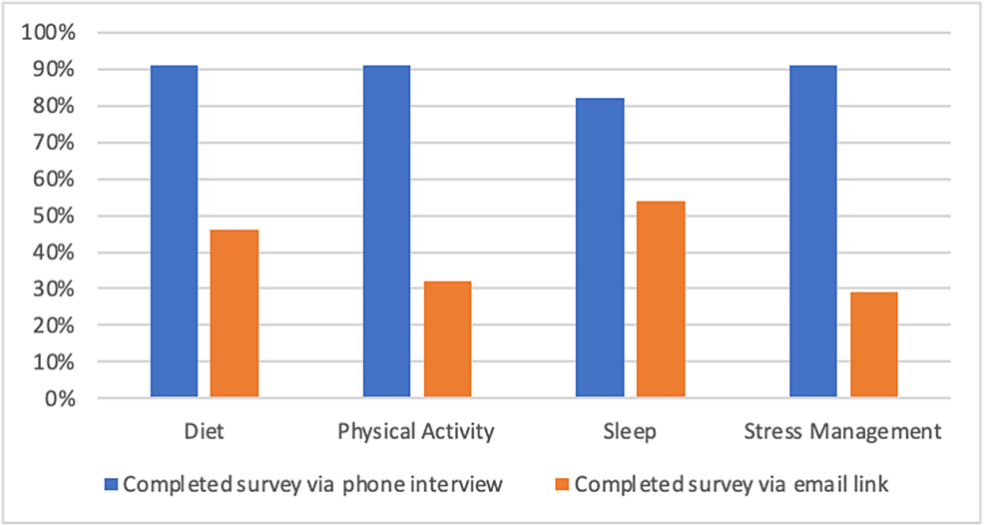
Percentage of clinics that indicated offering resources to facilitate health behavior change by survey completion method

Comparing the survey as completed via phone (22/50) versus email link (28/50), many more clinics claimed that they provided resources to patients to facilitate HBC when the survey was done over the phone (chart 4). For example, looking at surveys completed over the phone, about 91% (20/22) of these clinics indicated HBC resources or referral services in the domains of diet, physical activity, and stress management. However, looking at clinics who completed the survey via email link, only 13/28 (46%) offered resources for diet, 9/28 (32%) for physical activity, 15/28 (54%) for sleep, and 8/28 (28%) for stress management. This difference in offerings was statistically significant across all three domains: diet (p = 0.01), exercise (p = 0), and stress management (p = 0) (see Figure 4).

## Discussion

This study sheds light on the current practices of primary care and specialty clinics in Alabama regarding the facilitation of HBC in the domains of diet, physical activity, sleep, and stress management. Study results indicate that a significant percentage (34-44%) of primary care and specialty clinics do not offer HBC resources or referrals in these domains. More than a third of clinics indicated offering no resources or referrals for HBC in the domains of diet/eating patterns and sleep, and an even larger proportion (42-44%) indicated not doing so for physical activity and stress management. These findings underscore a gap in comprehensive lifestyle intervention support in primary care and specialty settings. 

Comparing these results with existing literature, it is evident that despite the well-documented benefits of HBC counseling in preventing and managing NCCDs [5-10], implementation remains inconsistent [21,23-25]. This inconsistency may be attributed to several barriers identified in previous studies, including limited time during patient visits [21-23], lack of training among PCPs [21,25,27,28], and misaligned incentives of current payment models [26]. For example, providers who implement lifestyle medicine within their practices face reimbursement barriers in the U.S.’s fee-for-service healthcare payment model. These barriers highlight the need for innovative solutions to integrate HBC counseling more effectively into routine primary care. The high likelihood of clinics referring patients to free virtual HBC programs, once available, signals an opportunity to leverage technology to overcome some of the barriers to providing HBC counseling. Virtual programs could offer a scalable and accessible solution, potentially enhancing patient engagement in lifestyle changes and improving health outcomes.

This study, while providing valuable insights into the practices of primary care and specialty clinics in Alabama regarding HBC facilitation, is not without limitations. First, the response rate (41%) represents a potential limitation. A low response rate may limit the generalizability of the study’s findings, as the views and practices of non-responding clinics are not represented. This raises questions about whether the clinics that chose to participate are systematically different from those that did not, potentially biasing results toward clinics more engaged in or aware of HBC practices. Second, response bias, particularly between responders and non-responders, and differences in responses based on survey completion method (email vs. phone) present another limitation. Study findings indicated a significant discrepancy in the reported provision of HBC resources between those who completed the survey over the phone and those who did so via email. This discrepancy suggests that the mode of survey completion may have influenced responses, with those completing the survey over the phone possibly subject to social desirability bias. Further, specific details regarding approaches utilized were not captured. For instance, it would be informative to know what provider counseling includes across clinics, what websites and handouts were utilized, etc. One potential way to address these limitations is to have medical students collect data regarding clinic approaches to facilitating HBC while on clinical rotations.

## Conclusions

This study, which has shown the lack of HBC resources offered in clinics within Alabama, highlights the crucial role that primary care and specialty clinics could have in facilitating HBC to combat NCCDs. Despite the recognized importance of addressing key lifestyle factors, such as diet, physical activity, sleep, and stress management, in managing NCCDs, there remains a gap in the consistent delivery of comprehensive HBC counseling and support. The findings suggest a readiness among clinics to refer patients to virtual HBC programs, indicating a promising avenue for expanding access to necessary interventions. To move forward, it is imperative for healthcare providers, administrators, and policymakers to prioritize the integration of HBC counseling into primary care. Emphasizing the development of accessible, scalable, and effective HBC interventions, particularly those leveraging virtual platforms, can significantly enhance patient care and outcomes. By addressing existing barriers and harnessing innovative solutions, clinics can better support patients in making sustainable lifestyle changes, ultimately reducing the prevalence and impact of NCCDs.
